# ECDomainMiner: discovering hidden associations between enzyme commission numbers and Pfam domains

**DOI:** 10.1186/s12859-017-1519-x

**Published:** 2017-02-13

**Authors:** Seyed Ziaeddin Alborzi, Marie-Dominique Devignes, David W. Ritchie

**Affiliations:** 1 Université de Lorraine, LORIA, UMR, Vandœuvre-lès-Nancy, 7503, 54506 France; 2 CNRS, LORIA, UMR, Vandœuvre-lès-Nancy, 7503, 54506 France; 3 Inria Nancy Grand-Es, Villers-lès-Nancy, 54600 France

**Keywords:** Content-based filtering, Protein domain, Protein function, Enzyme commission number, Pfam domain

## Abstract

**Background:**

Many entries in the protein data bank (PDB) are annotated to show their component protein domains according to the Pfam classification, as well as their biological function through the enzyme commission (EC) numbering scheme. However, despite the fact that the biological activity of many proteins often arises from specific domain-domain and domain-ligand interactions, current on-line resources rarely provide a direct mapping from structure to function at the domain level. Since the PDB now contains many tens of thousands of protein chains, and since protein sequence databases can dwarf such numbers by orders of magnitude, there is a pressing need to develop automatic structure-function annotation tools which can operate at the domain level.

**Results:**

This article presents ECDomainMiner, a novel content-based filtering approach to automatically infer associations between EC numbers and Pfam domains. ECDomainMiner finds a total of 20,728 non-redundant EC-Pfam associations with a F-measure of 0.95 with respect to a “Gold Standard” test set extracted from InterPro. Compared to the 1515 manually curated EC-Pfam associations in InterPro, ECDomainMiner infers a 13-fold increase in the number of EC-Pfam associations.

**Conclusion:**

These EC-Pfam associations could be used to annotate some 58,722 protein chains in the PDB which currently lack any EC annotation. The ECDomainMiner database is publicly available at http://ecdm.loria.fr/.

## Background

Proteins perform many essential biological functions such as catalysing metabolic reactions and mediating signals between cells. These functions are often carried out by distinct “domains”, which may be identified as highly conserved regions within a multiple alignment of a group of similar protein sequences, as in the Pfam classification [1]. It is widely accepted that such protein domains often correspond to distinct and stable three-dimensional (3D) structures, and that there is often a close relationship between protein structure and protein function [2]. Indeed, it is well known that protein structures are often more highly conserved than protein sequences [3], and this suggests that proteins with similar structures will have similar biological functions [4]. The Protein Data Bank (PDB) [5, 6] now contains over 107,000 3D structures, most of which have been solved by X-ray crystallography or NMR spectroscopy.

As well as sequence-based and structure-based classifications, proteins may also be classified according to their function. For example, the Enzyme Commission [7] uses a hierarchical four-digit numbering system to classify the enzymatic function of many proteins. The first digit, or top-level “branch” of the hierarchy, selects one of six principal enzyme classes (oxidoreductase, transferase, hydrolase, lyase, isomerase, and ligase). The second digit defines a general enzyme class (chemical substrate type). The third digit defines a more specific enzyme-substrate class (e.g. to distinguish methyl transferase from formyl transferase), while the fourth digit, if present, defines a particular enzyme substrate. However, it should be noted that because EC numbers are assigned according to the reaction catalyzed, it is possible for different proteins to be assigned the same EC number even if they have no sequence similarity or if they belong to different structural families.

**Fig. 1:**
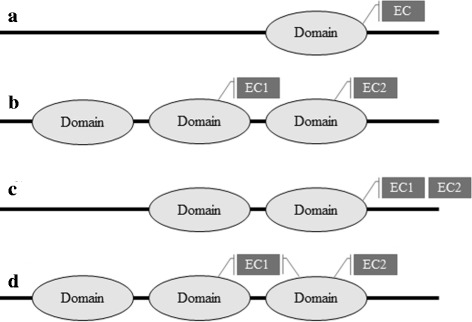
**a**) One domain provides one enzyme function; (**b**) two domains on the same chain each provide a different enzyme function; (**c**) one domain provides two different enzyme functions; (**d**) one domain provides one enzyme function, while a second domain acts as a co-factor with the first domain to provide an additional enzyme function

Furthermore, there are several ways in which a protein might provide one or more enzymatic functions, as illustrated in Fig. 1. In the simplest case (Fig. 1 a), a protein contains just one domain, and there is is a one-to-one association between that domain and a particular enzymatic function. In this case, it is reasonable to suppose that the catalytic site is located entirely on that domain. Similarly, a protein may have two or more distinct domains, each of which provides a distinct enzymatic (or non-enzymatic) function (Fig. 1 b). On the other hand, a protein domain could be involved in more than one catalytic activity, as illustrated in Fig. 1 c. Finally, a catalytic site may be at the interface between two domains, or one domain serves as a necessary co-factor for the other (Fig. 1 d). Clearly, it is biologically relevant to be able to distinguish all such cases. However, except for the simplest case (Fig. 1 a), it can be seen that finding domain-EC associations automatically is a non-trivial task. Several groups have described approaches or resources that can associate entire PDB protein chains with enzyme EC numbers [8–11]. Probably the most up-to-date and exhaustive association between PDB chains and EC numbers is provided by SIFTS [12], which is a collaboration between the Protein Data Bank in Europe and UniProt [13]. SIFTS incorporates a semi-automated procedure which links PDB chain entries to external biological resources such as Pfam, and IntEnz [14].

While all of the above mentioned approaches can provide associations between PDB protein chains and enzyme EC numbers, to our knowledge, very few approaches have been published for automatically assigning EC numbers to structural domains. SCOPEC [15] uses sequence information from SwissProt and PDB entries that have been previously annotated with EC numbers in order to assign EC numbers to SCOP domains [16]. It first looks for PDB chains that fully map to SwissProt entries (to within up to 70 residues) and that match on at least the first three EC number digits. In this way, SCOPEC identifies single domain structures that can be associated unambiguously with an EC number. Although SCOPEC can subsequently propagate a known EC-domain association to a matching domain in a multi-domain protein, it is generally not able to resolve cases where multiple ECs are associated with multi-domain chains (parts B, C, and D in Fig. 1. Furthermore, it appears that the SCOPEC database is no longer available on-line.

In contrast, the dcGO ontology database for protein domains produced in 2012 is still available online and provides several ontological annotations (Gene Ontology: GO, EC, pathways, phenotype, anatomy and disease ontologies) for more than 2000 SCOP domain families [17].

The dcGO approach follows the principle that if a GO term tends to be attached to proteins in UniProtKB that contain a certain domain, then that term should be associated with that domain. The statistical significance of an association is assessed against a random chance association using a hypergeometric distribution followed by multiple hypotheses testing in terms of false discovery rate. The dcGO approach addresses the issues of hierarchical structure of most biological ontologies and the nature of domain composition for multi-domain proteins. However, a mapping onto Pfam domains is proposed only for GO terms and not for EC numbers.

Here, we describe a recommender-based approach call “ECDomainMiner” for associating Pfam domains with EC numbers, which builds on our previously described statistical approach [18]. Recommender systems are a class of information filtering system [19, 20] which aim to present a list of items that might be of interest to an on-line customer. There are two main kinds of recommender systems. Collaborative filtering approaches make associations by calculating the similarity between activities of users [21, 22]. Content-based filtering aims to predict associations between user profiles and description of items by identifying common attributes [20, 23]. Such an approach has recently been applied to a quite different problem of discovering novel cancer drug combinations [24].

Here, we use content-based filtering to associate EC numbers with Pfam domains from existing EC-chain and Pfam-chain associations from SIFTS, and from EC-sequence and Pfam-sequence associations from SwissProt and TrEMBL, where protein chains and sequences serve as the common attributes through which EC-Pfam associations are made. Note that our approach *does not* attempt to identify catalytic sites or catalytic residues. Rather, we aim to detect frequent co-occurrences of Pfam domains and EC numbers in order to deconvolute the often complex EC-Pfam relationships within multi-domain and multi-function protein chains. We assess the performance of our approach against a “Gold Standard” dataset derived from InterPro [25], and we compare our results with the Pfam-EC associations derived from the dcGO database. We also show how our database of more than 20,000 EC-Pfam associations can be exploited for automatic annotation purposes.

## Methods

### Data preparation

Our data sources are SIFTS for EC number and Pfam domain annotations of PDB chains, and Uniprot for EC number and Pfam domain annotations of protein sequences. UniProt is divided into three parts: (i) the non-redundant, high quality, manually curated SwissProt part, (ii) the TrEMBL data that are annotated using Unified Rules [26], called here UniRule, and (iii) the rest called here TrEMBL.

In addition, in order to parameterise and evaluate ECDomainMiner, we use the InterPro database [25] which contains a large number of manually curated EC-Pfam associations. Flat data files of SIFTS (July 2015), Uniprot (July 2015), and InterPro (version 53.0) were downloaded and parsed using in-house Python scripts. From the SIFTS data, associations between EC numbers and PDB chains, and associations between PDB chains and Pfam domains were extracted. Associations between Uniprot sequence accession numbers (ANs) and EC numbers, and AN-Pfam associations were then extracted from the SwissProt section of Uniprot to give a dataset of Swissprot associations. For the TrEMBL entries, we collected and stored the corresponding AN-EC and AN-Pfam associations which had been annotated by UniRule, and those associations lacking UniRule annotations to give two further sequence-based datasets of associations, which we call the UniRule and TrEMBL association datasets.

To avoid bias due to duplicate structures or sequences in the four source datasets, all PDB chains and Uniprot sequences were grouped into clusters having 100% sequence identity using the Uniref non-redundant cluster annotations [27], and each cluster was assigned a cluster unique identifier (CID). Note that since just a few point mutations can dramatically change an enzyme’s substrate specificity, making clusters of identical rather than highly similar sequences avoids the risk of falsely clustering proteins that share highly similar folds but which have quite different substrates. The source EC-chain and EC-AN associations were then mapped to the corresponding CID in order to make four sets of EC-CID associations. A similar mapping was applied to the source Pfam-chain and Pfam-AN associations to give four sets of Pfam-CID associations.

For the reference data, we extracted from InterPro a total of 1515 EC-Pfam associations in which each EC number had all four digits and each Pfam accession number referred either to a Pfam domain or a Pfam family (i.e. Pfam motifs and repeats were excluded). These associations were considered to be “positive examples”, and were randomly divided into two equal “training” and “test” subsets. However, for training purposes, we also needed some “negative examples”. We therefore created a set of “false” EC-Pfam associations by first shuffling the CID-EC and CID-Pfam associations from SIFTS dataset, and by then randomly collecting 1515 wrong EC-Pfam associations from the shuffled datasets. In the rest of this article, we will refer to the combined set of 758 randomly chosen positive examples from InterPro and 758 randomly chosen negative examples as our “training dataset” and the remaining 1513 positive and negative examples as our “test dataset”.

### Inferring EC-Pfam domain associations

The main idea underlying the discovery of hidden EC-Pfam associations is to assign a feature vector to each EC number and each Pfam domain, where the length of the vector is given by the total number of PDB and UniProt CIDs, and where each vector element marks the existence (1) or absence (0) of an EC number or Pfam domain annotation for a particular CID. Each possible EC-Pfam association is then scored using the cosine similarity between the corresponding pair of EC and Pfam feature vectors.

**Fig. 2:**
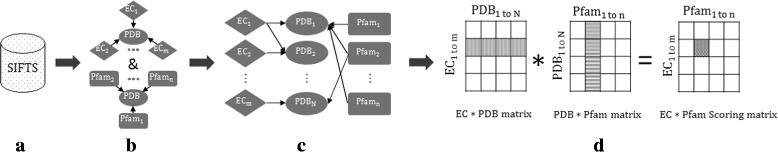
A graphical illustration of calculating raw EC-Pfam association scores from existing SIFTS EC-CID and Pfam-CID associations

The various steps of our content-based filter approach for finding associations between 4-digit EC numbers and Pfam domains are illustrated in Fig. 2 for the SIFTS dataset. First, all relations between PDB CIDs and EC numbers, and between PDB CIDs and Pfam domains are extracted from SIFTS, as described above. Joining these two lists of relations then yields a complex many-to-many graph that contains relations between EC numbers, PDB CIDs, and Pfam domains.

After this join operation, all EC-CID relations are encoded in a binary matrix, where a 1 represents the presence of an association and a 0 represents no association. This matrix is then row-normalised such that each row has unit magnitude when considered as a vector. Similarly, all PDB CID-Pfam relations are encoded in a second binary matrix which is column-normalised. Consequently, the product of the two normalised matrices corresponds to a matrix of cosine similarity scores between the rows of the first matrix and the columns of the second matrix. Thus, each element, *S*(*e* *c*,*d*), of the product matrix represents a raw association score between an EC number, *ec*, and a Pfam domain, *d*.

Similarly, raw EC-Pfam association scores are calculated from EC-CID and Pfam-CID relations extracted from SwissProt, TrEMBL and Unirule. Then, because we wish to draw upon the relations from all four datasets, we combine the four raw scores as a weighted average to give a single normalized confidence score, *C* *S*_*ec*,*d*_:

1 $$  {CS}_{ec,d} = \frac{\sum_{i} w_{i} S_{i}(ec,d)} {\sum_{i} w_{i}}  $$

where *i*∈{*S* *I* *F* *T* *S*,*S* *w* *i* *s* *s* *p* *r* *o* *t*,*T* *r* *E* *M* *B* *L*,*U* *n* *i* *R* *u* *l* *e*} enumerates the four datasets, *w*_*i*_ are weight factors, to be determined, and where an individual association score, *S*_*i*_(*e* *c*,*d*), is set to zero whenever there is no data for the (*e* *c*,*d*) pair in dataset *i*.

In order to find the best values for the four weight factors, receiver-operator-characteristic (ROC) curves [28] were calculated using the positive examples of our Interpro-based training dataset, against the remaining associations (background associations).

Each weight was varied from 0.0 to 1.0 in steps of 0.1, and for each combination of weights a ROC curve of the ranked association scores was calculated. The combination of weights that gave the largest area under the curve (AUC) of the ROC curve was selected.

### Defining a confidence score threshold

Having determined the best weight for each data source, we next wished to determine an overall threshold for the confidence score. To do this in an objective way, we scored and ranked the members of the training dataset, and labeled them true or false according to a threshold value that was varied from 0.0 to 1.0 in steps of 0.01. For each threshold value, we counted the number of positive examples above the threshold (TPs), negative examples above the threshold (FPs), negative examples below the threshold (TNs), and positive examples below the threshold (FNs). We then calculated the recall, *R*, precision, *P*, and their harmonic mean in order to obtain a “F-measure” using:

2 $$ R = \frac{TP}{TP + FN},\qquad P = \frac{TP}{TP + FP},\quad \quad F = \frac{2 R P}{P + R}.  $$

The score threshold that gave the best F-measure was checked on the test subset and selected as the best threshold to use for accepting inferred associations.

### Exploiting the EC number hierarchy

The above approach has focused on finding explicit co-occurrences between Pfam domains and 4-digit EC numbers. However, it is possible to find more associations by relaxing the criteria for co-occurrences of EC-Pfam annotations by looking for matches only at the 3-digit EC level. Indeed, we have observed several cases where true associations according to the InterPro training dataset were assigned confidence scores below the threshold value because they had too few (4-digit EC number) instances to provide sufficient support. Therefore, the above procedure was repeated using 3-digit EC numbers to give a 3-digit scoring scheme (with different weight factors and a different score threshold). Then, any 4-digit EC-Pfam association below the 4-digit threshold, but consistent with a 3-digit EC-Pfam association above the 3-digit threshold, was added to the final list of accepted 4-digit EC-Pfam associations. It should be clarified that “consistent” means here that the 4-digit EC number is a descendant of the 3-digit EC number and that the Pfam domains are the same.

### Hypergeometric distribution *p*-value analysis

While the above procedure provides a systematic way to infer EC-Pfam associations, we wished to estimate the statistical significance, and thus the degree of confidence, that might be attached to those predictions. More specifically, we wished to calculate the probability, or “*p*-value”, that an EC number and a Pfam domain might be found to be associated simply by chance. For example, it is natural to suppose such associations can be predicted at random if *ec* or *d* are highly represented in the structure/sequence CIDs. In principle, in order to estimate the probability of getting our EC-Pfam associations by chance, one could generate random datasets by shuffling the relations between EC numbers and CIDs on the one hand, and between Pfam domains and CIDs on the other hand. However, this is quite impractical given the very large numbers of CIDs, EC numbers, and Pfam domains, and the complexity of the filtering procedure that would have to be repeated for each shuffled version of the dataset. Therefore, as in [17], we assume that a random association of CIDs to pairs of *ec* and *d* follows a hypergeometric distribution.

Letting *N* denote the total number of CIDs, *N*_*d*_ the number of CIDs related to the Pfam domain *d*, and *N*_*ec*_ the number of CIDs related to the EC number *ec*, the hypergeometric probability distribution is given by

3 $$  p(X_{ec,d} \geqslant K_{ec,d}) = \frac{\sum_{i=K_{ec,d}}^{\min{(N_{d},N_{ec})}} {{{N_{ec}}\choose{i}}} {{{N-N_{ec}}\choose{N_{d}-i}}}} {{{N}\choose{N_{d}}}}, $$

where $p(X_{ec,d} \geqslant K_{ec,d})$ represents the probability of having a number *X*_*ec*,*d*_ equal to or greater than the observed number *K*_*ec*,*d*_ of CIDs associated with both *d* and *ec*. Traditionally, a *p*-value of less than 0.05 is taken to be statistically significant. However, because this test is applied to a large number of EC-Pfam associations, we apply a Bonferoni correction which takes into account the so-called family-wise error rate (FWER) [29]. We therefore consider any *p*-value less than 0.05/*T* as denoting a statistically significant inferred EC-Pfam association in a dataset, with *T* the total number of tested EC-Pfam associations for this dataset, In order to distinguish EC-Pfam associations using both confidence scores and *p*-values, we classify them into three classes, “Gold”, “Silver”, and “Bronze”. An association is assigned to the Gold class if both its EC-Pfam score is greater than the determined threshold and all its *p*-values (in all datasets) are statistically significant. An association is labeled Silver if its score is above the threshold but one or more of its *p*-values is not statistically significant, or if its score is below the threshold (due to the 3-digit procedure, see “Exploiting the EC number hierarchy” section) but all its *p*-values are statistically significant. All other associations are labeled Bronze.

## Results and discussion

### Data source weights and score threshold

After clustering identical structures and sequences, and calculating raw association scores (Fig. 2), our merged dataset contains 6306 SIFTS, 18,917 SwissProt, 124,699 TrEMBL, and 141,990 UniRule candidate EC-Pfam associations, giving a total of 262,571 distinct EC-Pfam associations to draw from Table 1. In our ROC-based training procedure, the best AUC value of 0.985 was obtained with weights *w*_*SIFTS*_=0.1, *w*_*SwissProt*_=1.0, *w*_*TrEMBL*_=0.1, and *w*_*UniRule*_=0.6. These weights clearly give greater importance to the candidate associations in SwissProt and UniRule, respectively, compared to those in SIFTS and TrEMBL.

**Table 1:** Statistics on the source datasets and calculated EC-Pfam associations

	Dataset	EC-Pfam associations	Distinct 4-digit EC numbers	Distinct Pfam entries
Source	SIFTS	6306	2648	2611
Datasets	SwissProt	18,917	4013	3101
	TrEMBL	124,699	3751	5703
	UniRule	141,990	1020	2907
	Merged	262,571	4648	6639
Reference	InterPro	1515	688	1284
ECDomainMiner	With CS above threshold	*8* *2* *5* *6*	*3* *7* *0* *1*	*3* *0* *2* *2*
Results	(Overlap with InterPro)	(*1* *4* *6* *1*)	(*6* *8* *8*)	(*1* *2* *4* *5*)
	Including low CS	*2* *0* *,* *7* *2* *8*	*4* *4* *5* *5*	*3* *6* *1* *3*
	(Overlap with InterPro)	(*1* *4* *9* *8*)	(*6* *8* *8*)	(*1* *2* *7* *3*)

CS is the Confidence Score

All italicized entries are calculated by ECDomainMiner

The optimal score threshold was determined according to the F-measure training procedure using our training dataset (“Defining a confidence score threshold” section). This gave a score threshold of 0.04 for a maximum F-Measure of 0.9476. Applying this threshold to the test dataset yielded a comparable F-measure of 0.935, and precision and recall values of 0.99 and 0.893, respectively.

### Global analysis of inferred EC-Pfam associations

The results of the ECDomainMiner approach are summarized in Table 1. This table shows the numbers of 4-digit EC-Pfam associations along with the numbers of distinct EC numbers and Pfam entries involved in those associations for the four sources and the merged datasets before filtering.

After applying the 0.04 score threshold, the number of EC-Pfam associations falls to 8,256 with an overlap of about 96% of InterPro reference associations. Using the relaxed 3-digit association approach (“Exploiting the EC number hierarchy” section), the final ECDomainMiner dataset contains 20,728 EC-Pfam associations that overlap by 99.3% the InterPro reference dataset. These numbers show that our approach efficiently retrieves the InterPro reference EC-Pfam associations, including a small percentage (about 3.3%) that have a low confidence score.

**Fig. 3:**
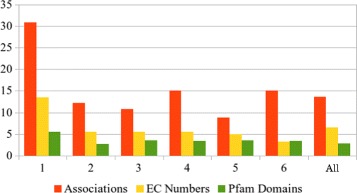
Scale-up factors for ECDomainMiner compared with InterPro. Ratios between the numbers in ECDomainMiner and in Interpro have been calculated for associations (*red*), EC numbers (*yellow*), and Pfam domains (*green*) after dividing the dataset according to each EC branch represented in the associations (1 to 6) and for all the dataset (All). 1: oxydoreductases; 2: transferases; 3: hydrolases; 4: lyases; 5: isomerases; 6: ligases

Table 1 also shows that our ECDomainMiner set of EC-Pfam associations represents a 13.7 fold-increase (20,728/1515) in EC-Pfam associations with respect to InterPro. Moreover, the list of EC-Pfam associations produced by ECDomainMiner contains 6.4 times more EC numbers and 2.8 times more Pfam domains than InterPro. Figure 3 shows how this increase in EC-Pfam associations distributes across the 6 top-level branches (i.e. 1-digit codes) of the EC classification.

The greatest ECDomainMiner scale-up factor occurs for associations involving the oxydoreductases (EC branch 1). The smaller scale-up factor observed for Pfam domains (2.8 versus 6.4 for EC numbers) can be explained by the fact that not all Pfam domains display an enzymatic activity. Thus there is a natural limit in the coverage of Pfam database by our EC-Pfam associations, whereas there is no such limit for the coverage of EC numbers. Combining the confidence scores with the calculated *p*-values as described in “Hypergeometric distribution *p*-value analysis” section gave 4552 Gold associations (having scores above the threshold and significant *p*-values in all source datasets), 11,426 Silver associations (with either scores above the threshold and one or more non-significant *p*-values, or with a score below the threshold but with significant *p*-values in all source datasets), and 4201 Bronze associations.

### Comparison with dcGO

**Fig. 4:**
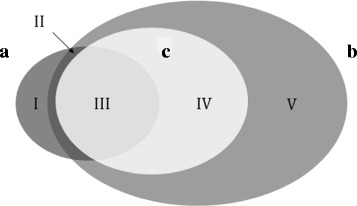
Venn diagram showing the intersection between **a** Pfam2EC (2500 associations) from dcGO, **b** All-Merged (262,571 associations), and **c** ECDomainMiner (20,728 associations). Region I (480 associations) is the portion of (**a**) for which there is no data in any of our four source datasets. Region II (128 associations) is the portion of (**a**) that exists in (**b**) but is not retained in ECDomainMiner (**c**). Region III (1892 associations) is the overlap between (**a**) and (**c**). Region IV (18,836 associations) is the portion of ECDomainMiner associations that are not available from SCOP2EC. Region V (241,363 associations) is the rest of the merged set of EC-Pfam source associations that are absent from (**a**) and not retained as Gold, Silver, or Bronze associations by ECDomainMiner

In order to compare ECDomainMiner with the dcGO approach [17], we extracted SCOP2EC associations from the Domain2EC file available from the dcGO database (http://supfam.org/SUPERFAMILY/dcGO). The Domain2EC file includes 7249 associations with 4-digit EC numbers, of which 3774 are related to SCOP “Families” and 3475 to SCOP “SuperFamilies”. Because InterPro only tabulates SCOP family domains, we limited our comparison to the set of 3774 SCOP2EC family associations. The SCOP families were mapped to Pfam families according to InterPro mapping files in order to generate a set of 2500 “Pfam2EC” associations (i.e. EC-Pfam associations which may be deduced directly from the SCOP2EC data). This set (shown as set a in Fig. 4) was compared with the set of all 262,571 merged EC-Pfam associations found by ECDomainMiner (set b in Fig. 4).

This comparison showed that a total of 480 Pfam2EC associations from SCOP2EC are not present in our merged dataset. The remaining 2020 Pfam2EC associations were then compared with the 20,728 associations calculated by ECDomainMiner (set c in Fig. 4). This comparison (the intersection of sets a and c) produced a total of 1892 EC-Pfam associations which are common to Pfam2EC and ECDomainMiner, indicating that ECDomainMiner agrees with 75.7% of the Pfam2EC associations from dcGO. Furthemore, this comparison also shows that ECDomainMiner result set contains 18,836 (20,728−1,892) additional EC-Pfam associations that are not available through dcGO.

### Selecting plausible associations in multi-domain proteins

Because ECDomainMiner finds many new EC-Pfam associations, it is important to ask to what extent it also might produce false associations. Firstly, we recall that ECDomainMiner eliminated more than 92% (241,843 out of 262,571) of low-scoring associations from the merged source dataset. This suggests that most of the eliminated associations involve Pfam domains that are not catalytically active. Indeed, if a Pfam domain is not regularly associated with protein chains or sequences having an enzymatic activity, the ECDomainMiner score for that domain is very low, and hence no EC number is assigned to that domain. This applies in particular to accessory domains that can co-occur with various catalytic domains in multi-domain proteins. A good example of such an accessory domain is PF00188 (the CAP protein family) which is a part of 216 different architectures. Among these architectures, there are 3 and 5 different architectures, which additionally contain PF00112 (Peptidase C1 domain) and PF00069 (Protein kinase domain), respectively. According to Pfam website, PF00188 is catalytically inactive but PF00112 and PF00069 are active. In fact, ECDomainMiner assigns PF00112 to 26 different EC numbers with a majority of EC 3.4.22 (Cysteine endopeptidases), and PF00069 to 28 different EC numbers that all start with 2.7 (Transferring phosphorus-containing groups). However, ECDomainMiner does not assign PF00188 to any EC number. This is because a large number of protein chains and sequences containing either PF00112 or PF00069 and associated with the above-mentioned EC activities, do not contain PF00188. In other words the catalytic activities of PF00112 and PF00069 are not strictly dependent on the presence of PF00188. Moreover, the SIFTS and UniProt databases indicate that PF00188 is associated with 43 different PDB chains and 5197 different protein sequences. However, none of those PDB chains are associated with an EC number in SIFTS and only 31 protein sequences (24 in TrEMBL and 7 in UniRule) are associated with at least one 4-digit EC number. Consequently, the association score of PF00188 with any EC number is zero for both the SIFTS and SwissProt datasets and is quite low (less than 0.02) for both the TrEMBL and UniRule datasets. Thus, the confidence scores of all of the associations involving PF00188 in ECDomainMiner are lower than our threshold of 0.04, and so these candidate associations are filtered out. This mechanism explains how an accessory domain is not assigned to an EC number by ECDomainMiner, and suggests that most of the retained associations are proper candidates for domain functional annotation.

### Single and multiple EC-Pfam associations

**Fig. 5:**
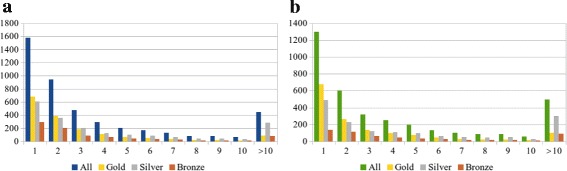
Distribution of EC numbers (**a**) and Pfam domains (**b**) in multiple associations. Numbers (1 to 10 and >10) represent the arity of the association in which a given EC number, respectively Pfam domain, is involved. In addition, for each arity, the normalized number of Gold, Silver, and Bronze associations is plotted. It can be observed that for arities equal to or greater than 4, the proportion of Silver associations is always the highest but significant numbers of Gold associations remain present even for high arity numbers

Exploring the ECDomainMiner results readily reveals that a given EC number or Pfam domain can be involved in one or more distinct EC-Pfam associations. Figure 5 shows the relative distribution of EC numbers and Pfam domains according to the number of EC-Pfam associations they are involved in. This figure shows that 1576 out of 4393 EC numbers and 1280 out of 3542 Pfam domains are involved in a single EC-Pfam association.

Although this represents rather high proportions of the total number of EC numbers and Pfam domains in ECDomainMiner (35.9 and 36.1%, respectively), the intersection of the concerned EC-Pfam single associations yields a list of only 97 one-to-one EC-Pfam associations, of which 62, 34, and 1 are Gold, Silver, and Bronze associations, respectively. Comparison with the InterPro reference dataset reveals that two thirds (65) of these one-to-one associations are novel compared to InterPro. Interestingly, we confirmed in our source datasets that all of these associations involve single-domain proteins. Thus, these unambiguous associations constitute the most reliable novel associations calculated by ECDomainMiner.

The complete list of one-to-one EC-Pfam associations found by ECDomainMiner may be downloaded from the ECDomainMiner web site. Interestingly 14 of these associations (8 Gold, of which 2 match InterPro reference associations, and 6 Silver) concern “DUF” (domain of unknown function) or “UPF” (uncharacterised protein family) Pfam entries. These are listed in part (A) of Table 2 in order of decreasing confidence score.

**Table 2:** (A) Fourteen one-to-one EC-Pfam associations found by ECDomainMiner and involving domains of unknown function, (B) an example of one-to-one EC-Pfam association with very similar EC and Pfam descriptions, and (C) two examples of obligate Pfam pairs associated with an EC number

	EC	Pfam	Score	EC name	Pfam name	Quality	PDBs (SIFTS)
A	2.7.8.28	PF01933	0.972	2-phospho-L-lactate transferase	Uncharacterised protein family UPF0052	Gold	9/0/11
	4.1.99.5	PF11266	0.944	Aldehyde oxygenase (deformylating)	Protein of unknown function DUF3066	Gold	18/0/0
	2.1.1.286	PF11968	0.889	25S rRNA (adenine(2142)-N(1))- methyltransferase	Putative methyltransferase DUF3321	Gold	0/0/0
	1.13.99.1	PF05153	0.667	Inositol oxygenase	Family of unknown function DUF706	Gold	4/0/0
	2.4.1.155	PF15027	0.611	Alpha-1,6-mannosyl-glycoprotein 6-beta-N-acetylglucosaminyltransferase	Domain of unknown function DUF4525	Gold	0/0/0
	4.2.3.130	PF10776	0.611	Tetraprenyl-beta-curcumene synthase	Protein of unknown function DUF2600	Gold	0/0/0
	2.3.1.78	PF07786	0.609	Heparan-alpha-glucosaminide N-acetyltransferase	Protein of unknown function DUF1624	Gold	0/0/0
	3.1.4.45	PF09992	0.584	N-acetylglucosamine-1-phosphodiester alpha-N-acetylglucosaminidase	Predicted periplasmic protein DUF2233	Gold	0/0/1
	1.13.12.20	PF08592	0.556	Noranthrone monooxygenase	Domain of unknown function DUF1772	Gold	0/0/0
	2.1.1.312	PF11312	0.556	25S rRNA (uracil(2843)-N(3))- methyltransferase.	Protein of unknown function DUF3115	Gold	0/0/0
	2.1.1.313	PF10354	0.556	25S rRNA (uracil(2634)-N(3))- methyltransferase	Domain of unknown function DUF2431	Gold	0/0/0
	2.5.1.128	PF01861	0.556	N4-bis(aminopropyl) spermidine synthase	Protein of unknown function DUF43	Gold	0/0/1
	5.2.1.14	PF13225	0.556	Beta-carotene isomerase	Domain of unknown function DUF4033	Gold	0/0/0
	1.14.99.29	PF04248	0.333	Deoxyhypusine monooxygenase	Domain of unknown function DUF427	Silver	0/0/5
B	6.3.2.25	PF03133	0.610	Tubulin–tyrosine ligase	Tubulin-tyrosine ligase family	Gold	0/2/21
C	$2.7.1.30\left \{{\vphantom {\frac {\frac {\frac {1}{1}}{1}}{1}}}\right.$	PF00370	0.847	Glycerol kinase	FGGY family of carbohydrate kinases, N-terminal domain	Gold	85/32/9
		PF02782	0.828		FGGY family of carbohydrate kinases, C-terminal domain	Gold	85/32/7
	$6.3.4.23 \left \{{\vphantom {\frac {\frac {\frac {1}{1}}{1}}{1}}}\right.$	PF06973	0.997	Formate-phosphoribosyl-amino- imidazol	DUF1297	Gold	16/3/0
		PF06849	0.997	carboxamide ligase	DUF1246	Gold	16/3/0

The ‘PDBs (SIFTS)’ column contains 3 counts of PDB chains containing the mentioned Pfam domain and having either the same EC annotation in SIFTS as calculated by ECDomainMiner (first position), or different EC annotations between SIFTS and ECDomainMiner (second position), or no EC annotations in SIFTS (third position). Complete lists of PDB identifiers may be retrieved from the ECDomainMiner web server

These examples demonstrate that ECDomainMiner can be used to enrich domain annotation. Visual inspection of the one-to-one EC-Pfam associations indicates that about one quarter of them (23) could have been retrieved simply by comparing the names associated with the EC number and the Pfam identifier, which are nearly identical (see example in Table 2b). However, only 10 of these associations were in fact already known in InterPro. Clearly, minor and unpredictable spelling differences impair the automatic retrieval of such similar but non-identical EC and Pfam names. Nonetheless, while these associations could be found by clever text matching, we emphasise that ECDomainMiner’s confidence scores and *p*-values provide a level of support for each association that would be very difficult to obtain from text mining alone.

The multi-partner associations calculated by ECDomainMiner provide many more complex EC-Pfam associations. As a first analysis of such multiple associations, we looked for obligate pairs or tuples of Pfam domains that are always associated with a given EC number. Briefly, for any pair of Pfam domains, (*d*_1_, *d*_2_), associated with the same EC number, *ec*, (i) we reject those pairs for which at least one *ec*-annotated CID (in any source dataset) occurs in relation with *d*_1_ and not *d*_2_ or with *d*_2_ and not *d*_1_, (ii) for all other pairs we calculate for each source dataset the ratio of the number of *ec*-annotated CIDs related to *d*_1_ and *d*_2_, to the total number of *ec*-annotated CIDs. A support ratio of 1 means that all CIDs annotated with *ec* in a dataset are also related to *d*_1_ and *d*_2_. A similar algorithm was used for triplets and quadruples of Pfam domains. For a support ratio of 1 in at least one source dataset, we found 907, 191 and 47 obligate associations between an EC number and a pair, a triplet or a quadruplet of Pfam domains. These associations are available from the ECDomainMiner website. Two examples are given in part (C) of Table 2.

Interestingly, filtering the names of the Pfam domains with the expressions “N-terminal” and “C-terminal” yielded 58 obligate pairs containing both a N-terminal and a C-terminal domain of the same function. This indicates that our approach is finding enzymes in which the catalytic function is provided by the interface between two consecutive Pfam domains. Only 4 of these obligate pair associations are currently documented in InterPro.

### Annotating PDB chains with EC numbers

Our analysis of the December 2015 release of the SIFTS database reveals that about 45% of PDB entries lack an EC number annotation. Of course, such an annotation is not expected to be present in all PDB entries because not all proteins have enzymatic activity. Nonetheless, it is interesting to use ECDomainMiner to analyse the number of PDB entries that contain Pfam domains which are present in EC-Pfam associations. Table 3 shows that a total of 58,722 PDB chains lacking EC annotations in SIFTS include at least one of the 3542 Pfam domains present in ECDomainMiner.

**Table 3:** The numbers of PDB protein chains that could be annotated by ECDomainMiner associations

Association type	ECDM associations concerned	PDB chains concerned
Any	14,573	58,722
Gold	3591	41,246
Silver	7796	44,406
Bronze	3186	34,820
One-to-One	44	1334

Overall, we calculated that these chains map to a total of 24,995 PDB entries that could benefit from the additional annotations inferred by ECDomainMiner. For those chains lacking EC annotations, ECDomainMiner finds Gold, Silver, and Bronze EC-Pfam associations for 41,246, 44,406 and 34,820 PDB chains, respectively. In particular, 1334 PDB chains could benefit from our dataset of 97 non ambiguous one-to-one EC-Pfam associations.

### The ECDomainMiner web server

The ECDomainMiner web server may be queried by EC number or Pfam domain. Thus, if one wishes to search for associations for a protein chain that currently lacks any EC annotation in the PDB (e.g. chain 2q7xA), one first needs to retrieve from the PDB the Pfam domain(s) that it contains (in this example, PF01933). Then, querying the ECDomainMiner server with each Pfam domain identifier will show the associated EC numbers (in this example, 2.7.8.28), along with the associated filtering scores and quality classes. In this example, ECDomainMiner finds a Gold quality association between PF01933, present in PDB chain 2q7xA, and EC number 2.7.8.28 (2-phospho-L-lactate transferase) which consequently can be associated with PDB entry 2q7x. Interestingly, PDB entry 2q7x is described as a putative phospho transferase from *streptococcus pneumoniae* tigr4, which is consistent with the enzymatic activity found by ECDomainMiner, and which could not be deduced from the Pfam domain name (UPF0052).

## Conclusion

We have presented a content-based filtering approach for associating EC numbers with Pfam domains. This approach has been shown to be able to infer a total of 20,728 non-redundant EC-Pfam associations, which corresponds to over 13 times as many EC-Pfam associations as currently exist in InterPro. Furthermore, thanks to our calculated *p*-values, we have assigned an intuitive quality rating (Gold, Silver, or Bronze) to each EC-Pfam association found. These calculated associations are publicly available on the ECDomainMiner web site. We anticipate that our content-based filtering approach may be applied to other annotation vocabularies or ontologies, and we are currently working to extend our approach to discover new GO-Pfam annotations.

We believe that enriching protein chain annotations will facilitate a better understanding and exploitation of structure-function relationships at the domain level. While many of the associations calculated by ECDomainMiner are consistent with those recently made available by the domain-centric dcGO approach for finding EC-SCOP associations, the ECDomainMiner results set contains many more associations than dcGO. Indeed, the ECDomainMiner result set contains 18,836 EC-Pfam which are not available in dcGO. Our analysis of the simple one-to-one associations found by ECDomainMiner shows that several DUF or UPF entries in Pfam may be assigned functions from the EC classification, and that obvious inconsistencies in the annotation texts may easily be corrected or unified. However, only a relatively small number (less than 0.5%) of EC-Pfam associations in our result set are simple one-to-one associations, indicating that there exist a large number of many-to-many relations between EC numbers and Pfam domains. Further analyses of these complex associations using graph database and machine-learning techniques could reveal many more hidden protein structure-function relationships.

## Abbreviations

AN: Accession numbers
AUC: Area under the curve
CID: Cluster unique IDentifier
CS: Confidence score
dcGO: Domain centric Gene Ontology database
DUF: Domain of unknown function
EC: Enzyme commission
FN: False negative
FP: False positive
GO: Gene ontology
NMR: Nuclear magnetic resonnance
P: Precision
PDB: Protein data bank
Pfam: Protein family database
R: Recall
ROC: Receiver operator characteristics
SCOP: Structural classification of proteins
SCOPEC: a database of catalytic domains
SIFTS: Structure integration with function, taxonomy and sequence
TN: True negative
TP: True positive
TrEMBL: Translated sequences from the European molecular biology laboratory bank
UniProtKB: UniProt knowledge base
UPF: Uncharacterized protein family

## References

[1] Finn RD, Bateman A, Clements J, Coggill P, Eberhardt RY, Eddy SR, Heger A, Hetherington K, Holm L, Mistry J, Sonnhammer ELL, Tate J, Punta M (2014). Pfam: the protein families database. Nucleic Acids Res.

[2] Berg JM, Tymoczko JL, Stryer L (2002). Protein structure and function.

[3] Chothia C, Lesk AM (1986). The relation between the divergence of sequence and structure in proteins. EMBO J.

[4] Martin ACR, Orengo CA, Hutchinson EG, Jones S, Karmirantzou M, Laskowski RA, Mitchell JBO, Taroni C, Thornton JM (1998). Protein folds and functions. Structure.

[5] Bernstein FC, Koetzle TF, Williams GJB, Meyer EF, Brice MD, Rodgers JR, Kennard O, Shimanouchi T, Tasumi M (1977). The protein data bank. Eur J Biochem.

[6] Gutmanas A, Alhroub Y, Battle GM, Berrisford JM, Bochet E, Conroy MJ, Dana JM, Montecelo MAF, van Ginkel G, Gore SP, Haslam P, Hatherley R, Hendrickx PMS, Hirshberg M, Lagerstedt I, Mir S, Mukhopadhyay A, Oldfield TJ, Patwardhan A, Rinaldi L, Sahni G, Sanz-García E, Sen S, Slowley RA, Velankar S, Wainwright ME, Kleywegt GJ (2014). PDBe: protein data bank in europe. Nucleic Acids Res.

[7] Webb EC (1992). Enzyme nomenclature 1992. recommendations of the nomenclature committee of the international union of biochemistry and molecular biology on the nomenclature and classification of enzymes.

[8] Reichert J, Jabs A, Slickers P, Sühnel J (2000). The IMB Jena image library of biological macromolecules. Nucleic Acids Res.

[9] de Beer TAP, Berka K, Thornton JM, Laskowski RA (2014). PDBsum additions. Nucleic Acids Res.

[10] Laskowski RA (2001). PDBsum: summaries and analyses of PDB structures. Nucleic Acids Res.

[11] Martin ACR (2004). PDBSprotEC: a web-accessible database linking PDB chains to EC numbers via SwissProt. Bioinformatics.

[12] Velankar S, Dana JM, Jacobsen J, van Ginkel G, Gane PJ, Luo J, Oldfield TJ, O’Donovan C, Martin MJ, Kleywegt GJ (2013). SIFTS: structure integration with function, taxonomy and sequences resource. Nucleic Acids Res.

[13] The UniProt Consortium (2010). The universal protein resource (UniProt) in 2010. Nucleic Acids Res.

[14] Fleischmann A, Darsow M, Degtyarenko K, Fleischmann W, Boyce S, Axelsen KB, Bairoch A, Schomburg D, Tipton KF, Apweiler R (2004). IntEnz, the integrated relational enzyme database. Nucleic Acids Res.

[15] George RA, Spriggs RV, Thornton JM, Al-Lazikani B, Swindells MB (2004). SCOPEC: a database of protein catalytic domains. Bioinformatics.

[16] Murzin AG, Brenner SE, Hubbard T, Chothia C (1995). SCOP: a structural classification of proteins database for the investigation of sequences and structures. J Mol Biol.

[17] Fang H, Gough J (2013). dcGO: database of domain-centric ontologies on functions, phenotypes, diseases and more. Nucleic Acids Res.

[18] Alborzi SZ, Devignes MD, Ritchie DW. EC-PSI: associating enzyme commission numbers with Pfam domains. In: Proceedings of JOBIM: 2015. doi:10.1101/022343.

[19] Hanani U, Shapira B, Shoval P (2001). Information filtering: Overview of issues, research and systems. User Model User-Adap Inter.

[20] Ricci F, Rokach L, Shapira B (2011). Introduction to recommender systems handbook.

[21] Breese JS, Heckerman D, Kadie C (1998). Empirical analysis of predictive algorithms for collaborative filtering. Proceedings of the Fourteenth Conference on Uncertainty in Artificial Intelligence.

[22] Koren Y, Bell R. Advances in collaborative filtering on recommender systems handbook. New York: Springer: 2015. p. 77–118.

[23] Basu C, Hirsh H, Cohen W (1998). Recommendation as classification: Using social and content-based information in recommendation. Proceedings of IAAI.

[24] Huang L, Li F, Sheng J, Xia X, Ma J, Zhan M, Wong ST (2014). Drugcomboranker: drug combination discovery based on target network analysis. Bioinformatics.

[25] Mitchell A, Chang HY, Daugherty L, Fraser M, Hunter S, Lopez R, McAnulla C, McMenamin C, Nuka G, Pesseat S, Sangrador-Vegas A, Scheremetjew M, Rato C, Yong SY, Bateman A, Punta M, Attwood TK, Sigrist CJA, Redaschi N, Rivoire C, Xenarios I, Kahn D, Guyot D, Bork P, Letunic I, Gough J, Oates M, Haft D, Huang H, Natale DA, Wu CH, Orengo C, Sillitoe I, Mi H, Thomas PD, Finn RD (2015). The InterPro protein families database: the classification resource after 15 years. Nucleic Acids Res.

[26] Pedruzzi I, Rivoire C, Auchincloss AH, Coudert E, Keller G, De Castro E, Baratin D, Cuche BA, Bougueleret L, Poux S (2013). Hamap in 2013, new developments in the protein family classification and annotation system. Nucleic Acids Res.

[27] Suzek BE, Huang H, McGarvey P, Mazumder R, Wu CH (2007). UniRef: comprehensive and non-redundant UniProt reference clusters. Bioinformatics.

[28] Fawcett T (2006). An introduction to ROC analysis. Pattern Recogn Lett.

[29] Cui X, Churchill GA (2003). Statistical tests for differential expression in cDNA microarray experiments. Genome Biol.

